# Self-Healing, Self-Adhesive and Stable Organohydrogel-Based Stretchable Oxygen Sensor with High Performance at Room Temperature

**DOI:** 10.1007/s40820-021-00787-0

**Published:** 2022-01-29

**Authors:** Yuning Liang, Zixuan Wu, Yaoming Wei, Qiongling Ding, Meital Zilberman, Kai Tao, Xi Xie, Jin Wu

**Affiliations:** 1 State Key Laboratory of Optoelectronic Materials and Technologies and the Guangdong Province Key Laboratory of Display Material and Technology, School of Electronics and Information Technology, Sun Yat-Sen University, Guangzhou, 510275 People’s Republic of China; 2 Department of Biomedical Engineering, Faculty of Engineering, Tel Aviv University, 69978 Tel Aviv, Israel; 3 Ministry of Education Key Laboratory of Micro and Nano Systems for Aerospace, Northwestern Polytechnical University, Xi’an, 710072 People’s Republic of China

**Keywords:** Stretchable oxygen sensors, Organohydrogel, Self-healing, Self-adhesive, Electrochemical reaction

## Abstract

With the advent of the 5G era and the rise of the Internet of Things, various sensors have received unprecedented attention, especially wearable and stretchable sensors in the healthcare field. Here, a stretchable, self-healable, self-adhesive, and room-temperature oxygen sensor with excellent repeatability, a full concentration detection range (0-100%), low theoretical limit of detection (5.7 ppm), high sensitivity (0.2%/ppm), good linearity, excellent temperature, and humidity tolerances is fabricated by using polyacrylamide-chitosan (PAM-CS) double network (DN) organohydrogel as a novel transducing material. The PAM-CS DN organohydrogel is transformed from the PAM-CS composite hydrogel using a facile soaking and solvent replacement strategy. Compared with the pristine hydrogel, the DN organohydrogel displays greatly enhanced mechanical strength, moisture retention, freezing resistance, and sensitivity to oxygen. Notably, applying the tensile strain improves both the sensitivity and response speed of the organohydrogel-based oxygen sensor. Furthermore, the response to the same concentration of oxygen before and after self-healing is basically the same. Importantly, we propose an electrochemical reaction mechanism to explain the positive current shift of the oxygen sensor and corroborate this sensing mechanism through rationally designed experiments. The organohydrogel oxygen sensor is used to monitor human respiration in real-time, verifying the feasibility of its practical application. This work provides ideas for fabricating more stretchable, self-healable, self-adhesive, and high-performance gas sensors using ion-conducting organohydrogels.

**Supplementary Information:**

The online version contains supplementary material available at 10.1007/s40820-021-00787-0.

## Highlights

- The organohydrogel-based O_2_ sensor features full concentration detection range (0-100%), ultralow limit of detection (5.7 ppm), high sensitivity (0.2%/ppm), excellent selectivity, tunable response/recovery speeds, good linearity, and room-temperature operation.
- The oxygen sensor can work normally under various extreme environmental conditions, such as low (below −18 °C) and high (above 40 °C) temperatures, dry (11.3% RH), and humid (90.5% RH) environments.
- An electrochemical reaction-based mechanism is proposed to elucidate the oxygen sensing behavior of ion-conducting organohydrogel.

**Supplementary Information:**

The online version contains supplementary material available at 10.1007/s40820-021-00787-0.

## Graphical abstract

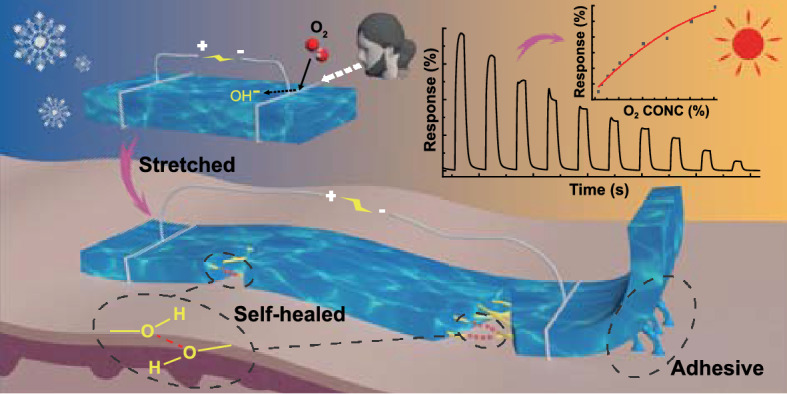

## Introduction

In recent years, with the rapid development of the Internet of Things (IoT) and the growing requirements of people’s life quality, various sensors used for monitoring health and environmental quality have gradually entered people’s daily lives to enhance people’s living standards [1–5]. For instance, gas sensors can be exploited to monitor the content of various gases in the environment [6–9]. Oxygen is the most indispensable gas in human life activities. It participates in the aerobic decomposition of carbohydrates that provide energy for life activities [10]. A low-oxygen environment will cause many adverse physiological reactions to the human body. Specifically, when the oxygen concentration is lower than 17%, it will cause symptoms such as dyspnea, fatigue, and decreased attention. Furthermore, suffocation fatal accidents may even occur when the oxygen concentration is lower than 12% [11, 12]. Otherwise, people need oxygen with a concentration higher than that in the air in some specific occasions, such as anesthesia machines and hyperbaric oxygen chambers in hospitals, and oxygen content analysis for aerospace and diving operations [13–15]. Therefore, the monitoring of oxygen with a wide range of concentrations is essential [16, 17]. In particular, if the oxygen sensor is stretchable, portable, self-healing, and self-adhesive, it can be directly attached to people’s clothing and even skin for persistent monitoring and can resist general mechanical damage. This will increase the convenience and prolong the service life of the sensor, which can greatly meet people's daily use requirements for wearable applications [18–21].

However, the gas sensing materials of conventional stretchable gas sensors cannot be stretched, self-healed, and self-adhesive. Instead, these stretchable gas sensors are fabricated by integrating non-flexible gas sensing materials, such as MoS_2_, V_2_O_5_, TiO_2_, and graphene, on flexible substrates, such as polydimethylsiloxane (PDMS), ecoflex, and Polyimide (PI) [22–27]. This will complicate the fabrication process. Furthermore, the stretchability of these sensors will also be limited by the substrates [28]. Most importantly, the detection objects of currently emerging stretchable gas sensors focus on toxic gases, such as NO_2_, SO_2_, and NH_3_, leaving stretchable oxygen sensors undeveloped [16, 29–31]. Among traditional oxygen sensors, people have the most in-depth research on chemiresistor-type sensors based on metal oxide semiconductors (MOS), which are easy to manufacture and therefore cost-effective [32, 33]. However, except for the lack of stretchability and self-healability, these sensors also have the problem of high operating temperature (100–500 °C). Furthermore, it is also difficult for them to achieve a wide detection range, good linearity, and fast response/recovery speeds [34–36]. For example, Xiong et al. prepared an oxygen sensor based on LaOCl-doped SnO_2_, which has the advantages of working at room temperature (RT) and high selectivity against H_2_, CH_4_, NH_3_, and CO_2_. But its detection range is limited within 100–5000 ppm, and the response and recovery speeds are slow. Specifically, the response and recovery time to 250 ppm O_2_ are 182 and 1315 s, respectively [37]. Although Xu et al. proposed the utilization of two-dimensional Bi_2_O_2_Se to detect O_2_ with an extremely low concentration of 0.25 ppm, the sensor failed to display the flexibility/stretchability, linearity, and fast response speed [38]. In addition, some oxygen sensors based on new sensing materials also have limitations [39, 40]. For example, Yeon Hoo Kim and co-workers proposed the utilization of MoS_2_ nanoparticles to prepare an oxygen sensor, which presented high and linear responses to O_2_ with a broad range of concentrations. But the sensor needs to be operated at 300 °C [12]. Until now, stretchable, self-healing, self-adhesive, and high-performance (linear, a wide detection range, selectivity, etc.) oxygen sensor working at RT has not yet been reported.

**Fig. 1:**
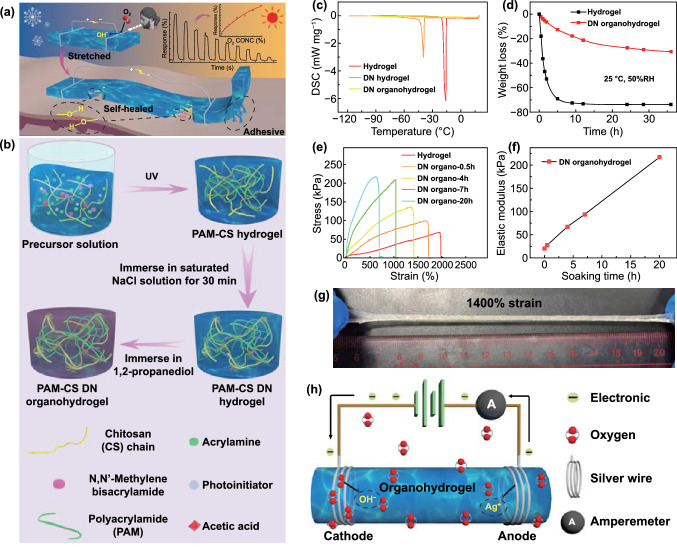
**a** Schematic illustrating the organohydrogel-based stretchable, self-adhesive, self-healable oxygen sensors working at room temperature. **b** Scheme showing the synthesis of PAM-CS DN organohydrogel. **c** DSC curves of hydrogel, DN hydrogel, and DN organohydrogel. **d** Time evolution of the weight loss percentage of hydrogel and DN organohydrogel when stored at 25 ℃ and 50% RH. **e** Stress-strain curves of hydrogel and DN organohydrogels obtained by soaking in 1,2-PD for 0.5, 4, 7, and 20 h, respectively. **f** Plot of the elastic modulus of DN organohydrogel vs soaking time. **g** Photograph showing the DN organohydrogel at 1400% tensile strain. **h** Schematic illustrating the working principle of the oxygen sensor

Hydrogel is a polymer material with a three-dimensional polymer network structure formed by chemical or physical cross-linking and swelled in a large amount of water. Many hydrogels have excellent stretchability, ionic conductivity, self-healability, biocompatibility, etc., and have been widely applied to fabricate wearable sensors for the monitoring of various physical and chemical quantities, such as pressure, temperature, strain, humidity, and the like [41–44]. Herein, for the first time, we employ polyacrylamide-chitosan (PAM-CS) composite organohydrogel to fabricate stretchable, self-healable, self-adhesive, and high-performance electronic oxygen sensors that can work at RT (27 °C) (Fig. 1a). Compared with traditional oxygen sensing materials, the organohydrogel features high stretchability (up to 1400% strain), self-healability, and RT operation. Furthermore, its good self-adhesive performance enables it to be directly adhered to clothing or even people’s skin for operation without entailing additional adhesive tape, which greatly simplifies the actual fabrication process of the wearable sensor. In addition, a facile soaking and solvent replacement strategy were devised to convert the hydrogel to corresponding double network (DN) organohydrogel with enhanced mechanical strength and moisture retention ability. 1,2-Propanediol (1,2-PD) was chosen for the solvent replacement instead of common glycerol owing to its smaller molecule and better permeability [45]. Thus, it should provide a more efficient solvent replacement process. More importantly, the effect of 1,2-PD on the moisture retention and freezing resistance of hydrogel has rarely been studied. The DN organohydrogel-based oxygen sensor also features full concentration detection range, low limit of detection (LOD), good linearity (within 0-20% O_2_), high sensitivity, and selectivity. Besides, the sensor can also operate properly in the stretched state and after self-healing, which is not available for previously reported oxygen sensors.

## Experimental Section

### Synthesis of Hydrogel and DN Organohydrogel

All chemicals including acrylamide (AM), N,N'-Methylene bisacrylamide (MBA), Irgacure2959 (photoinitiator), chitosan (degree of deacetylation > 90%, MW = 30,000), acetic acid, and 1,2-propanediol (1,2-PD) were purchased from Sigma-Aldrich. The PAM-CS composite hydrogel was prepared via a one-pot sol-gel process. Firstly, chitosan (0.8 g) and acetic acid (115 μL) were added to deionized water (20 g) and stirred magnetically at a speed of 900 rpm for 3 h, so that the chitosan was fully dissolved in water to form a chitosan solution. Then, AM (3.6 g), MBA (0.002 g), and photoinitiator (0.115 g) were added to the chitosan solution and stirred magnetically for 2 h to obtain a precursor solution, which formed PAM-CS composite hydrogel after being radiated with ultraviolet (UV) light for 1 h. The hydrogel was soaked in a saturated NaCl solution for 30 min to make the chitosan chains form a chain entanglement structure, generating the PAM–CS DN hydrogel. Finally, the prepared DN hydrogel was soaked in 1,2-PD for 10 h for the solvent replacement, leading to the formation of PAM-CS DN organohydrogel (Fig. 1b).

### Material Characterization

The differential scanning calorimetry (DSC) spectra were obtained on a Netzsch DSC-204. The Fourier transform infrared (FTIR) spectra were acquired on a Thermo Scientific Nicolet 6700 FTIR Spectrometer. Field emission scanning electron microscopy (SEM) (ZEISS SUPRA 60) was deployed to analyze the surface morphology and elements of the electrodes. The Instron machine (S6566) was employed to acquire the strain–stress curves and the peel force vs displacement curves.

### Gas Sensing Characterization

A homemade gas sensing characterization system was deployed for the gas sensing test at 27 °C and 74% relative humidity (RH) if without special indication. An airtight bottle with an air inlet and outlet was utilized as the gas chamber. The sensor was placed in the test chamber, and the Ag wires were deployed as the electrodes of the sensor and led out of the gas chamber. The Keithley 2400 source meter was exploited to monitor the current in the circuit after a constant DC bias voltage of 0.5 V was applied. The DIG-III gas distribution system was utilized to control the flow rate of two gas streams, one of which was the mixture of O_2_ and N_2_, and the other was pure N_2_. The two gas streams were mixed with different ratios to obtain specific concentrations of O_2_. During the test, pure N_2_ was employed to purge the test chamber before and after feeding O_2_. To measure the gas sensing properties of sensor at different strains, two binders were utilized to fix the stretched organohydrogel on a glass slide after applying certain tensile strains such as 25%, 50%, and 100%.

## Results and Discussion

### Characterization of Organohydrogel

The highly stretchable PAM-CS hydrogel was synthesized via a facile one-pot sol-gel process. The details are described in the experimental part. Specifically, the hydrogel was soaked in saturated sodium chloride (NaCl) solution to enable the dispersed chitosan chains to form a chain entanglement structure in the first step, thereby converting the PAM-CS composite hydrogel into a DN hydrogel. Then the DN hydrogel was soaked in 1,2-PD to replace part of the water in the solvent with 1,2-PD, leading to the formation of PAM-CS DN organohydrogel with a water-1,2-PD binary solvent. By comparing the FTIR spectra of hydrogel and DN organohydrogel, it can be seen that the intensities of the O–H stretching peak at 3347 cm^−1^ and O–H bending peak at 1040 cm^−1^ of the DN organohydrogel are higher than that of corresponding hydrogel (Fig. S1). This is attributed to the introduction of 1,2-PD molecules in the hydrogel through the solvent replacement strategy, which is also confirmed by the DSC measurement (Fig. 1c). The DSC spectra show that, compared with the freezing point of untreated hydrogel (-16 °C), the DN hydrogel obtained by the soaking strategy displays a much lower freezing point (-40 °C). The freezing point of the untreated hydrogel is lower than 0 °C. This is because the functional groups such as amino and hydroxyl groups on the polymer chain can form hydrogen bonds with water molecules, which increases the content of bound water and thus lowers the freezing point [46, 47]. The decrease in the freezing point of DN hydrogel is attributed to the formation of a chitosan chain entanglement structure with the aid of NaCl, enhancing the stability of the structure. Furthermore, the incorporation of NaCl also decreases the freezing point due to the ionic hydration effect. On this basis, the anti-freezing performance of the DN organohydrogel has been further boosted by using the solvent replacement strategy to incorporate 1,2-PD in the solvent, as the freezing point has dropped to below -120 °C. It is because each 1,2-PD molecule contains two hydroxyl groups, which can form strong hydrogen bonds with water molecules, which inhibits the formation of ice crystals at subzero temperatures [48].

Hydrogels are generally prone to lose water, which greatly affects the stability of both hydrogels and related devices. In particular, most gas sensors need to be directly exposed to air, making it difficult for hydrogel-based gas sensors to be practically applied, especially for long-term stable applications [49]. Fortunately, the drying resistance of hydrogel can be greatly enhanced by creating DN organohydrogel, in addition to the anti-freezing capacity [50]. Specifically, when the hydrogel was exposed to a dry environment of 24 °C and 50% RH for 5 h, its mass loss reached as high as 70%. By comparison, the mass loss of DN organohydrogel stabilized at about 30% within 36 h under the same circumstance, implying the boosted water retention ability of organohydrogel (Fig. 1d). Comparing the states of hydrogel and DN organohydrogel before and after the drying experiment, it can be found that after 36 h, the hydrogel has completely turned into a hard block and cannot bear deformations such as stretching, while the DN organohydrogel can still withstand 250% tensile strain (Fig. S2). It verifies that the introduction of 1,2-PD in the solvent can significantly improve the moisture holding capability of the hydrogel. This is because 1,2-PD introduces a large number of hydroxyl groups, which increase the content of bound water by forming strong hydrogen bonds with water molecules [51].

Moreover, compared with the untreated hydrogel, the mechanical strength of DN organohydrogel soaked in NaCl solution and 1,2-PD can be remarkably promoted at the expense of certain stretchability (Fig. 1e). Furthermore, the longer the hydrogel was soaked in 1,2-PD, the greater its elastic modulus (Fig. 1f). Specifically, the elastic modulus of organohydrogel increased from 20.3 to 208.2 kPa with prolonged immersion time from 0 to 20 h, showing 10.26 times enhanced mechanical strength. This is because the hydroxyl groups of 1,2-PD molecules formed a large number of hydrogen bonds with the hydroxyl or amino groups of the chitosan chains, which greatly strengthened its originally loose chain entanglement structure. Considering the balance between the stretchability and mechanical strength, the best formulation can be obtained by using the soaking time of hydrogel in NaCl solution and 1,2-PD of 0.5 and 7 h, respectively. With this approach, the obtained DN organohydrogel displays the elastic modulus of 94 kPa, and meanwhile can withstand more than 1400% tensile strain (Fig. 1g). In addition to the high toughness, both the hydrogel and DN organohydrogel can withstand severe deformations, such as 180° bending and 720° twisting deformations (Fig. S3).

### Gas sensing Mechanism and Performance

A two-electrode chemiresistor-type structure was employed to construct the oxygen sensor, in which the organohydrogel and the silver wires at both ends functioned as the solid-state electrolyte and electrodes, respectively, as shown in Fig. 1h. The gas sensing performance of oxygen sensors was characterized by monitoring the change of current flowing through the organohydrogel under a constant direct-current (DC) voltage of 0.5 V when exposed to pure nitrogen (N_2_) and specific concentrations of oxygen alternately. We propose the redox reactions occurring at the hydrogel-electrode interface to explain the gas responsive behaviors of the organohydrogel sensor. The entire system can be regarded as an electrolytic cell. When the organohydrogel is in a pure N_2_ environment, the steady-state current flowing through it is I_0_. When the sensor is exposed to a test gas containing a specific concentration of oxygen, the oxygen gains electrons at the cathode, and a reduction reaction occurs, leading to the generation of OH^−^:

1 $${\text{O}}_{2} + 2{\text{H}}_{2} {\text{O}} + 4e^{ - } \to 4{\text{OH}}^{ - }$$

Meanwhile, the silver electrode loses electrons at the anode, and an oxidation reaction occurs to generate Ag^+^:

2 $${\text{Ag}} - e^{ - } \to {\text{Ag}}^{ + }$$

3 $${\text{Ag}}^{ + } + {\text{Cl}}^{ - } \to {\text{AgCl}}$$

**Fig. 2:**
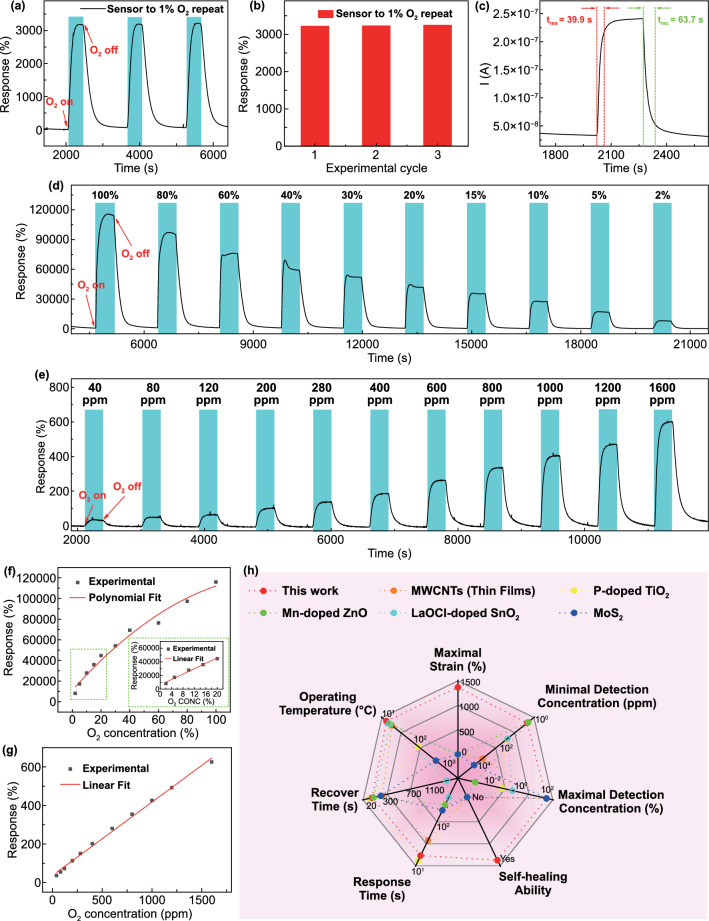
O_2_ sensing properties of the organohydrogel sensor. **a** Time-dependent response of the sensor to 1% O_2_ in three experimental cycles. The blue and white areas represent the “on” and “off” states of test gas in the gas chamber. **b** Quantitative response extracted from **a** versus experimental cycle. **c** Investigation of the response and recovery time in the detection of 1% O_2_. **d**, **e** Time-dependent responses of the sensor upon exposure to O_2_ with wide ranges of high (2-100%) and low (40-1600 ppm) concentrations, respectively. **f** The polynomial fit of responses versus O_2_ concentration within 2%-100%. The green box in the inset shows a linear fit of response versus O_2_ concentration from 2 to 20%. **g** The linear fit of responses versus O_2_ concentration within 40-1600 ppm. **h** The radar chart comparing the performance of this O_2_ sensor with that of other O_2_ sensors

The response here is defined as ${(I}_{{O}_{2}}-{I}_{0})/{I}_{0}$, where ${I}_{{O}_{2}}$ and ${I}_{0}$ are the stabilized currents when the sensor is exposed to O_2_ and pure N_2_, respectively. It is found that the contact surface area has little effect on the magnitude of response of the sensor. This is because the larger contact surface area enabled by increasing the coiling number of the Ag wire promotes both the electrochemical reactions and the initial current (baseline of the response curve), leading to the increase in both ${I}_{{O}_{2}}$ and ${I}_{0}$ (Fig. S4). The two effects offset each other, leading to a similar response. Figure 2a shows the dynamic response curve of the sensor upon exposure to pure N_2_ and 1% O_2_ repeatedly and alternately. It can be seen that the sensor produced a constant and high response of 3200–1% O_2_, indicating its excellent repeatability (Fig. 2b). The hysteresis curves of the sensor are shown in Fig. S5. When the oxygen concentration increases from 1 to 16% and then decreases to 1% at the same rate, the calibration curves in the ascending and descending O_2_ concentrations are highly similar. As such, the sensor displays a low hysteresis of 0.4%, which also proves its good reproducibility. The response time is defined as the time taken for 90% of the total current variation after a specific concentration of O_2_ is continuously introduced; while the recovery time is denoted as the time required to recover to 10% of the original current after removing O_2_ in the chamber using N_2_ purge [52]. As such, the response and recovery time in the detection of 1% O_2_ are 39.9 and 63.7 s, respectively (Fig. 2c). Compared with previously reported oxygen sensors based on other sensing materials, such as multi-walled carbon nanotubes (MWCNTs), ZnO, TiO_2_, and SnO_2_, the response and recovery time of the PAM–CS DN organohydrogel O_2_ sensors are equivalent or shorter (Table S1).

Figure S6a shows the dynamic curve of current versus time when the sensor was exposed to 2%-100% O_2_, and the corresponding dynamic response curve of the sensor is shown in Fig. 2d. It can be observed that when the sensor was exposed to O_2_, the current through it increased immediately. Furthermore, the higher the O_2_ concentration, the greater the current through the sensor, and thus the greater response obtained. It demonstrates the capability of the organohydrogel sensor to discriminate different O_2_ concentrations within a wide range. This is because O_2_ participated in an electrochemical reaction at the electrode of the sensor, which generated a Faraday current. The higher the oxygen concentration, the more O_2_ was involved in the electrochemical reaction, and the greater the Faraday current generated, leading to a higher response. Since the test time is as long as 6 h, the weight loss of the organohydrogel itself may also decrease the performance of the oxygen sensor. To eliminate this interference and get a rigorous conclusion, a durability test was also conducted, which was to expose the sensor to pure N_2_ and 1% O_2_ repeatedly and alternately for as long as 6 h (Fig. S6b). It can be seen that the response of the sensor to 1% O_2_ is relatively stable within 6 h with an error of less than 4%, which proves the durability of the organohydrogel oxygen sensor during long-term testing. In addition, the sensor can maintain a relatively stable current when exposed to the air for more than 8 h, which further verifies its good stability (Fig. S6c).

In addition to high O_2_ concentrations, the sensor also showed appreciable responses to low concentrations of O_2_ (40–1600 ppm), as shown in Fig. 2e. Moreover, there is a strong positive correlation between the response and oxygen concentration within 2-100% O_2_ (Fig. 2f). Notably, there is a significant linear relationship between the response and O_2_ concentration within 2-20% (the green box in the inset of Fig. 2f). Figure 2g further depicts that the sensor maintained good linearity at low O_2_ concentrations (40–1600 ppm). Note that 40 ppm O_2_ is the low-limit O_2_ concentration provided by our current experimental setup, but can be well perceived by this sensor. The good linearity within a wide range of O_2_ concentrations is advantageous for practical application. The slope of response versus concentration curve within 0-20% O_2_ was calculated as 0.2%/ppm, which was the sensitivity. The low theoretical LOD of 5.7 ppm O_2_ was further obtained by calculating the root mean square deviation of the noise from the dynamic response curve and the sensitivity (Fig. S7a-b, Table S2) [53]. In general, the oxygen sensor features excellent repeatability, a full concentration detection range, linearity within 0-20% O_2_, high sensitivity (0.2%/ppm), and low LOD (5.7 ppm), and therefore compares advantageously with existing oxygen sensors (Fig. 2h, Table S1).

### Self-Healing and Self-Adhesive Properties

**Fig. 3:**
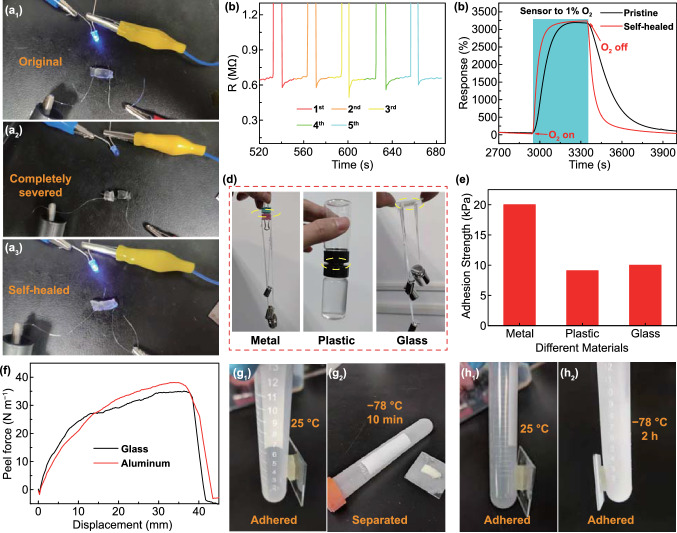
Self-healing and self-adhesive abilities of organohydrogel. **a** The change of on and off states of the LED indicator in the series circuit when the organohydrogel was in the states of (1) original, (2) completely severed, and (3) self-healed. **b** Time evolution of cyclical electrical self-healing processes of organohydrogels by real-time resistance measurement. **c** Dynamic responses of the sensor to 1% O_2_ in the pristine and self-healed states. **d** Photographs showing the organohydrogel (marked by the yellow circle) adhered firmly to various materials: metals, plastic, and glass. **e** The adhesion strength of organohydrogel on different substrates. **f** Peel force versus displacement curves of organohydrogel when adhered on different substrates. Photographs depicting **g** the untreated hydrogel could and could not adhere to the plastic centrifuge tube at 25 and -78 °C, respectively, whereas **h** the organohydrogel adhered firmly to the plastic centrifuge tube at both temperatures

In actual wearable applications, the sensor may be suffered from external forces and break. Fortunately, the self-healing ability of the organohydrogel can perfectly solve this problem, which is a unique advantage of the organohydrogel-based sensor [48, 54]. To investigate the electrical self-healability of organohydrogel, the organohydrogel, a blue LED indicator, and a 3 V DC voltage source were connected in series to form a circuit (Fig. 3a). The LED indicator was lighted before cutting off the conductive organohydrogel, while went out after being completely severed. Notably, the LED indicator was lightened again after reconnection of the cut organohydrogel at RT, suggesting the ready self-healability of the electrical conductance*.* To further quantitatively characterize the self-healing efficiency of the organohydrogel, the Keithley 2400 was used to measure the resistance of the organohydrogel before and after the disconnection and self-healing (Fig. 3b). It was found that the resistance of the organohydrogel returned quickly to the original level after self-healing, illustrating that the repeated disconnection and reconnection of the organohydrogel at RT did not impair the electrical conductivity, reflecting the high self-healing efficiency. It is because the disconnected organohydrogel can utilize many dynamic hydrogen bonds formed between hydroxyl and amino groups on the chitosan chain and the PAM chain to reconnect the polymer chains, which helps restore its conductivity and some mechanical properties. To further test the self-healability in the gas sensing properties, we cut off the organohydrogel and then contacted the cuts to make it heal naturally. The response of the organohydrogel sensor to 1% O_2_ after self-healing was about 3170%, which was basically the same as that before disconnection (3200%) (Fig. 3c). To better recover its mechanical properties, after the cuts of the broken organohydrogel were brought to contact with each other, they were wrapped with a tin foil and then heated at 95 °C for 10 min. The organohydrogel repaired by the heating method could still be stretched to 250% strain without the appearance of obvious gaps, while the naturally repaired organohydrogel would break again when it was subjected to about 100% strain (Fig. S8a-b). Moreover, the elastic modulus of the organohydrogel repaired by heating is 289 kPa, which is higher than that of the natural repaired counterpart (170 kPa). The increased healing efficiency after heating may be related to the accelerated movement of polymer chains, which promotes the reconnection of polymer chains. The readily self-healed electrical, gas sensing, and mechanical properties of the organohydrogels are conducive to long-term and durable wearable applications [55, 56].

The self-adhesive properties enable hydrogels to be directly adhered to various materials without requiring external adhesives, which is essential for a number of emerging applications, such as wearable sensors and bioelectronics, although self-adhesive gas sensor has seldom been reported [57]. Hydrogels generally achieve strong adhesion through the synergy of chemical bonds, the topology of connection, and mechanics of dissipation [58, 59]. Note that chitosan contains numerous amino and hydroxyl groups. Furthermore, both PAM and 1,2-PD contain abundant hydroxyl groups. Therefore, the PAM-CS DN organohydrogel is a strong hydrogen-bonded complex with excellent self-adhesive ability on different materials [60]. As shown in Fig. 3d, two metal clips were firmly glued together by the organohydrogel, and the lower metal clip could bear a weight of 80 g. Furthermore, two plastic bottle caps could also be glued together by the organohydrogel. The glass bottle below was filled with water and weighed 40 g in total. In addition, two glass slides were glued by the organohydrogel, and the lower glass sheet could withstand a weight of 100 g. Notably, the organohydrogel displays varied adhesion strength on different materials. Specifically, the adhesion strengths on glass, plastic, and metal (aluminum) were 20, 9.1, and 10 kPa, respectively (Fig. 3e). To further evaluate the adhesive properties, the peel strength of organohydrogel to various substrates was measured using the 180° peeling experiment (Fig. S9a). Figure 3f shows the peel force vs displacement curves of organohydrogel on glass and aluminum sheets, from which the peel strength of organohydrogel on both glass and aluminum sheets was determined as 35 N m^−1^.

Although both the hydrogel and organohydrogel exhibited good adhesion at RT, the organohydrogel with a water-diol binary solvent displayed much better self-adhesive ability at low temperatures, e.g., -78 °C due to its excellent anti-frost capacity. After the untreated hydrogel and organohydrogel had adhered to the plastic centrifuge tube, they were stored in a low-temperature box (-78 °C). It was found that the untreated hydrogel completely froze into ice and thus separated from the tube after 10 min, while the organohydrogel showed no signs of freezing after 2 h and therefore could still firmly adhere to the tube (Fig. 3g, h). It is because the untreated hydrogel has a freezing point of -16 °C, and a large part of the water molecules in the solvent freezes at a low temperature, causing the phase separation between polymers and solvents [61]. While the freezing point of the organohydrogel is below -120 °C, so there will be no phase separation between the polymer and the solvent. At this time, numerous hydrogen bonds inside can still function normally at a low temperature of -78 °C, making the organohydrogel maintain excellent self-adhesiveness. These results collectively demonstrate the excellent self-adhesive properties of DN organohydrogel within a wide range of temperatures, which allows the organohydrogel sensor to be directly attached to people's clothing to monitor the ambient oxygen concentration without the demand for additional adhesive items, such as tape (Fig. S9b). Even when the clothing was highly deformed, no detachment between the sensor and the clothing was observed, manifesting the great promise of the adhesive sensor for wearable application.

### Gas Sensing Properties under Different Deformations, Humidity, and Temperatures

**Fig. 4:**
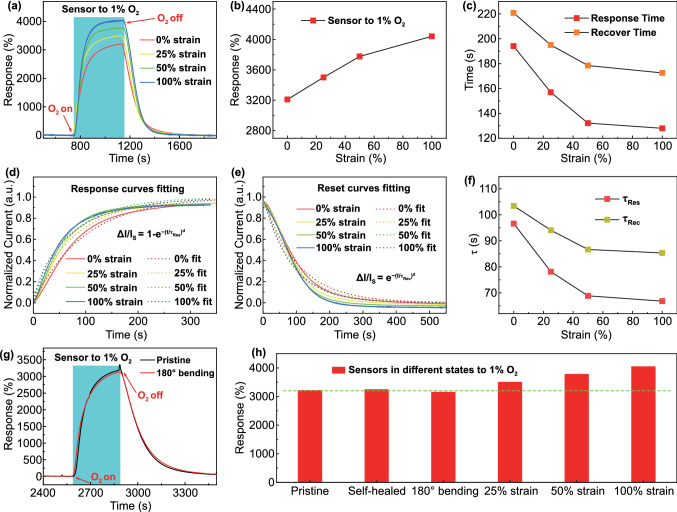
Influence of deformation on O_2_ sensing properties of organohydrogel sensor.** a** Dynamic response curves to 1% O_2_ at 0%, 25%, 50%, and 100% tensile strains. **b** Response extracted from **a** versus strain. **c** Response and recovery time t_90_ extracted from **a** versus strain. **d** Normalized current variations in response to 1% O_2_ at different strains with fits to the exponential growth formula. **e** Normalized current variations in the recovery process with fits to the exponential decay formula at different strains. **f** Plots of time constants (${\uptau }_{Res}$ and ${\uptau }_{Rec}$) versus tensile strain. **g** Real-time responses to 1% O_2_ in pristine and 180° bending states. **h** Quantitative responses to 1% O_2_ in the states of pristine, self-healed, 180° bending, 25%, 50%, and 100% tensile strains

Compared with traditional O_2_ sensors, this organohydrogel O_2_ sensor provides the unique capability to work under various mechanical deformations, such as stretching and bending (Fig. 4). Furthermore, such large deformation as 100% tensile strain did not degrade the gas sensing performance, but rather improved both the sensitivity and response/recovery speeds. Specifically, the greater the tensile strain applied to the sensor, the higher its response to oxygen, and meanwhile the shorter the response and recovery time. Also, when the 100% tensile strain was applied to the sensor, the response of the sensor to 1% O_2_ increased from 3210 to 4041%, suggesting an increment of 26%. The response and recovery time of the sensor were reduced from 194 and 221 s to 128 and 172 s, respectively, depicting a reduction of 34% and 22%, respectively (Fig. 4a-c). Possibly this is because the baseline of response curve before exposure to oxygen was declined due to the increased resistance of organohydrogel with strain (Fig. S10), whereas the electrons produced or consumed at the hydrogel-electrode interface due to electrochemical reactions were unchanged, leading to the increased relative current variation (response). Furthermore, both the internal structure and the surface area of the organohydrogel were changed during stretching. Specifically, the curly polymer chains straightened, and the pores in the organohydrogel might become larger, which promoted the diffusion of oxygen into the organohydrogel. In addition, the surface area of organohydrogel increased with strain, enhancing the adsorption of oxygen. Different from the freestanding organohydrogel as used for the gas sensing in Fig. 2c, the stretched organohydrogel was attached on a glass slide for studying the impact of strain on the gas sensing characteristics (Fig. S10a). Since the gas adsorption at the organohydrogel-glass interface was blocked in this case, it took a longer time to complete the gas diffusion and reaction.

To further research on the change of response/recovery time with strain, the dynamic response curves of the sensor under different tensile strains were fitted according to the exponential growth formula [62, 63]:

4 $$\Delta I/I_{S} = 1 - e^{{ - (t/\tau_{{{\text{Res}}}} )^{d} }}$$

where ${I}_{S}$ is the saturation current, ${\tau }_{Res}$ is the response time constant, $d$ is an exponent between 0 and 1. Figure 4d shows the experimental result and fitting curves at different strains. From the exponential fitting, the response time constants ${\tau }_{Res}$ of 96.6, 78.1, 68.8, and 66.8 s were obtained at 0%, 25%, 50%, and 100% strains, respectively, with the exponent $d$ of 0.99, 0.92, 0.93, and 0.82, respectively. Similarly, the dynamic reset curves were fitted according to the exponential decay formula:

5 $$\Delta I/I_{S} = e^{{ - (t/\tau_{{{\text{Rec}}}} )^{d} }}$$

where ${\tau }_{Rec}$ is the recovery time constant, which is 103.4, 94.1, 86.7, and 85.3 s at 0%, 25%, 50%, and 100% strains, respectively (Fig. 4e). It is clear that the greater the tensile strain, the smaller the ${\tau }_{Res}$ and ${\tau }_{Rec} (\mathrm{Fig}. 4\mathrm{f})$, which is consistent with the change of response and recovery time with strain as illustrated in Fig. 4c.

The stretchable organohydrogel sensor also displayed the clear electromechanical response, which was the change of resistance with tensile strain. It can be seen that the resistance augmented linearly with tensile strain from 0 to 200%, from which the gauge factor (GF) of 3.24 was derived (Fig. S10b-c). This suggests the possibility of employing the organohydrogel sensor to detect multiple stimuli. The remarkable electromechanical response of the sensor is attributed to the geometric variation of organohydrogel. As long as the response of the oxygen sensor in various tensile states is tested in advance, the interference of tensile strain to gas sensing can also be eliminated through the calibration with an external strain sensor in the practical wearable application. Except for tensile strain, other deformations such as bending and twisting hardly affect the resistance and response of the sensor. Notably, the excellent self-adhesive properties of the organohydrogel enabled the sensor to be bent for 180° and directly stuck on the glass slide to investigate its gas sensing characteristics at a highly bent state without using external adhesive (Fig. S11). Figure 4g shows that the response of the sensor to 1% O_2_ before and after bending was basically the same, i.e., ~ 3200%. Therefore, the response of the sensor was maintained whether it was deformed or after being broken and self-healed naturally (Fig. 4h). These attributes not only make the sensor very suitable for the application in wearable electronics but also extend its life span [64].

**Fig. 5:**
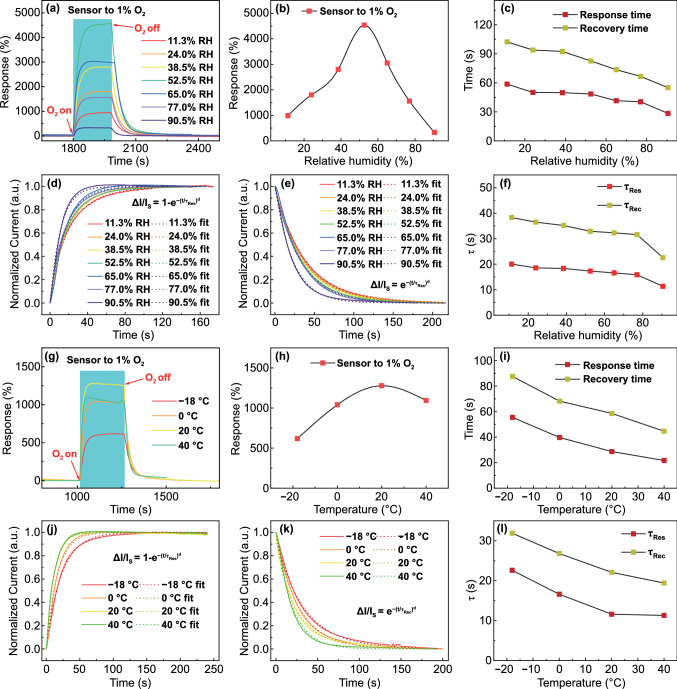
Influences of humidity and temperature on O_2_ sensing properties of organohydrogel sensor.** a** Dynamic response curves to 1% O_2_ under 11.3%, 24.0%, 38.5%, 52.5%, 65.0%, 77.0%, and 90.5% RH. **b** Response extracted from **a** versus relative humidity. **c** Response and recovery time t_90_ extracted from **a** versus relative humidity. **d** and **e** Normalized current variations in response to 1% O_2_ under different RH with fits to the exponential growth formula in the response and recovery processes, respectively. **f** Plots of time constants (${\uptau }_{Res}$ and ${\uptau }_{Rec}$) versus relative humidity. **g** Dynamic response curves to 1% O_2_ at -18, 0, 20, and 40℃. **h** Response extracted from **g** versus temperature. **i** Response and recovery time t_90_ extracted from **g** versus temperature. **j** and **k** Normalized current variations in response to 1% O_2_ under different temperatures with fits to the exponential growth formula in the response and recovery processes, respectively. **l** Plots of time constants (${\uptau }_{Res}$ and ${\uptau }_{Rec}$) versus temperature

Notably, both humidity and temperature are important influencing factors for room-temperature gas sensors. In actual wearable applications, the humidity and temperature of the environment where different people are located are different, which requires the organohydrogel oxygen sensor to work normally under various humidity and temperatures. Figure 5a, b shows the responsive behavior of the sensor to 1% O_2_ under different RH. Obviously, when the RH is about 52.5%, the sensor displays the highest response to oxygen, which is 4538%. When the RH is higher or lower than 52.5%, the response shows a downward trend. It is because the content of water molecules in the environment is overly low when the RH is low, which is unfavorable to the progress of the reduction reaction at the cathode, resulting in a decrease in the redox response. However, the organohydrogel absorbs superabundant water molecules by itself when the RH is high, thereby increasing its conductance and raising the baseline of the response curve before exposure to oxygen. This becomes the main factor affecting the response, leading to a decrease in the relative current variations. In addition, the response and recovery time of the sensor will decrease as the RH increases, which dropped from 58.7 s and 102.3 s under 11.3% RH to 28.4 s and 55 s under 90.5% RH, respectively (Fig. 5c). This is also due to the increase in the content of water molecules in the environment, which increases the rate of redox reactions. Similar to the study of the influence of tensile strain on the response/recovery speeds, the dynamic response and reset curves of the sensor under different RH were fitted according to the exponential growth formula, and ${\tau }_{Res}$ and ${\tau }_{Rec}$ were extracted (Fig. 5d–f). Clearly, with the increase in RH, ${\tau }_{Res}$ and ${\tau }_{Rec}$ decrease from 20 and 38.3 s under 11.3% RH to 11.3 s and 22.6 s under 90.5% RH, respectively, which are the same as the changing trend of response and recovery time in Fig. 5c.

In addition, the responsive behavior of the sensor to 1% O_2_ at different temperatures was investigated. The result in Fig. 5g-h shows that the sensor has an optimal working temperature, which is about 20 °C. When the temperature is higher or lower than 20 °C, the response will also show a downward trend. The low response at -18 °C is because the low temperature is unfavorable for the progress of electrochemical reactions [65]. However, when the sensor is at a high temperature, the baseline of the response curve will also raise due to the thermally activated ionic migration, which becomes the main factor affecting the response, resulting in a decrease in the relative current variation [66]. As with humidity, the response and recovery time of the sensor will decrease with increasing temperature, which dropped from 55.5 and 87.6 s at -18 °C to 21.7 s and 44.6 s at 40 °C, respectively (Fig. 5i). This is because the rise in temperature increases the rate of electrochemical reactions, which in turn increases the response/recovery speeds. Fitting the response and reset curves with the exponential decay formula allows for the extraction of the ${\tau }_{Res}$ and ${\tau }_{Rec}$ (Fig. 5j, k). Their tendency to change with temperature is the same as that of the response and recovery time, which decreases from 22.6 and 31.9 s at -18 °C to 11.3 s and 19.4 s at 40 °C (Fig. 5l).

Therefore, the best working temperature and humidity of the organohydrogel oxygen sensor are 20 °C and 52.5% RH, respectively, which is the comfort zone in our daily life. Note that the sensor can also work normally no matter in low/high temperature, dry/wet environments, or under different tensile strains. Just like eliminating the interference of tensile strain on the sensor, calibrating the organohydrogel oxygen sensor by integrating temperature and humidity sensors can also eliminate the interference of them. The capability to work normally within a broad range of temperature and humidity not only greatly expands the application scope of the oxygen sensor, but also makes it applicable to wearable fields and increases the value of its practical application.

### Investigation of the Sensing Mechanism

**Fig. 6:**
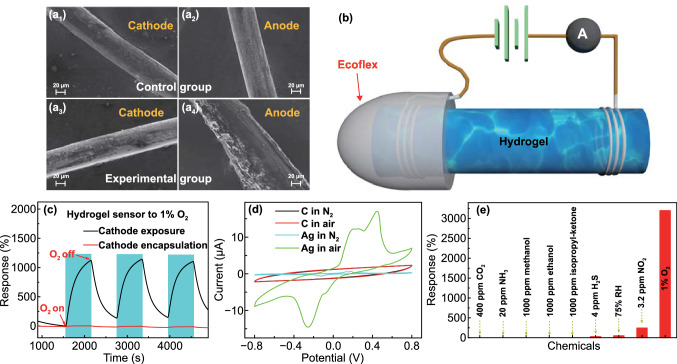
**a** SEM images of (1) cathode and (2) anode in the control group, and (3) cathode and (4) anode in the experimental group after exposure to air for 5 h. **b** Schematic illustrating the device configuration in the cathode encapsulation experiment. **c** Dynamic responses of the sensor to 1% O_2_ before and after encapsulation of cathode. **d** Current-Potential curves of the sensor when Ag and carbon (C) electrodes were employed in N_2_ and air, respectively. **e** Comparison in the responses of the sensor to 400 ppm CO_2_, 20 ppm NH_3_, 4 ppm H_2_S, 3.2 ppm NO_2_, and 1% O_2_

To confirm the aforementioned electrochemical gas sensing mechanism of organohydrogel sensor (Fig. 1h), we designed a series of experiments. First, we exploited SEM and its energy dispersive spectroscopy (EDS) module to investigate the variations of surface morphology and composition of electrodes before and after the gas sensing test using a high DC voltage (5 V). Two pieces of organohydrogel prepared in the same batch were connected with Ag wires, which functioned as the electrodes of the sensors when the DC voltage was and was not applied in the experimental and control groups, respectively, for 5 h. Both of the two sensors were exposed to the air (with 20.9% O_2_). After 5 h, the surface morphology and composition of anode and cathode materials (Ag wires) in the experimental and control groups were compared (Figs. 6a, S12, and Table 1). For the experimental group, the anode Ag was oxidized to form AgCl, while the cathode Ag kept intact. It verified that oxygen was reduced at the cathode, while the cathode metal itself was not consumed in the reaction. For the control group, neither the morphology nor the composition variation was observed for both the anode and cathode materials. It confirms the aforementioned redox reaction-based gas sensing mechanism.

**Table 1:** EDS element analysis of the cathode and anode in the control and experimental groups after exposure to O_2_ for 5 h

Groups (%)	Electrode	Element	Atomic percentage
Control	Cathode	Ag	100
	Anode	Ag	100
Experimental	Cathode	Ag	100
	Anode	Ag	39.43
		Cl	46.4
		Na	14.17

Note that we have also specially devised a cathode encapsulation experiment to further certify the proposed sensing mechanism. Specifically, the response of the sensor to 1% O_2_ was compared before and after the cathode was encapsulated by an Ecoflex film with enough thickness to isolate it from outside air (Fig. 6b). Before the Ecoflex precursor solidified, the cathode of the sensor was immersed in it. After the polymerization of Ecoflex, the cathode of the sensor was naturally encapsulated (Fig. S13). The anode of the sensor was normally exposed during the sensing test. Figure 6c shows that the response of the sensor to the same concentration of oxygen dropped significantly, close to 0, when the cathode was encapsulated, manifesting that the oxygen reacted at the cathode-organohydrogel interface. This also explains the phenomenon of positive current displacement of the sensor in response to oxygen.

To further explore the redox reactions occurring at the interface, the cyclic voltammetry (CV) curves of the sensor under N_2_ and air environment were scanned when Ag and carbon electrodes were applied. It can be seen from Fig. 6d that the CV curves measured by the carbon electrodes in both N_2_ and air environment basically coincided, implying that no redox peak appeared on the CV curves. This is because the carbon electrodes cannot be oxidized by O_2_, and thus the sensor cannot undergo an electrochemical reaction, demonstrating the important role of electrodes in the gas sensing of ion-conducting organohydrogel. The sensor based on Ag electrodes exhibited only two very small redox peaks (caused by the redox reactions of trace O_2_ dissolved in organohydrogel) in a N_2_ environment, while two obvious redox peaks appeared in air (resulting from the oxidation of Ag electrode by O_2_). This experiment consolidated the electrochemical reaction-based gas sensing mechanism from another perspective.

The internal three-dimensional polymer network structure of PAM-CS hydrogel allows it to retain a large amount of water in a solid form, and both the hydroxyl and the amino groups of chitosan can form hydrogen bonds with H_2_O and O_2_ to promote their adsorption. All of these provide favorable conditions for the full progress of electrochemical reactions. In addition, the incorporation of 1,2-PD in the organohydrogel brings a large number of hydroxyl groups, which can also form hydrogen bonds with H_2_O and O_2._ It not only improves the moisture retention of organohydrogel but also promotes the adsorption of reactants, which is conducive to the proceeding of electrochemical reactions. Figure S14 shows the response of the organohydrogel sensor to 1% O_2_ is significantly greater than that of the hydrogel sensor, which may stem from the lower current baseline of the organohydrogel sensor and the enhanced ability of organohydrogel to adsorb both H_2_O and O_2_ by readily forming hydrogen bonds between these molecules and 1,2-PD. Apart from O_2_, the sensor showed negligible responses to other oxidizing or reducing gases or other gaseous chemicals, such as CO_2_, NH_3_, methanol, ethanol, isopropyl-ketone, H_2_S, humidity, and NO_2_ (Figs. 6e and S15). For instance, the response of the sensor to 4 ppm H_2_S is only 29%, which is two orders of magnitude lower than that to 1% O_2_. Although its response to 3.2 ppm NO_2_ can reach 240%, the sensor cannot detect the ppm level of NO_2_ in an air environment, owing to the high response and high concentration of oxygen (Fig. S16). Moreover, the content of these gases in the common environment is extremely low compared with that of O_2_. Therefore, the interference from these gases is negligible, reflecting the high selectivity of the O_2_ sensor.

### Practical Applications

**Fig. 7:**
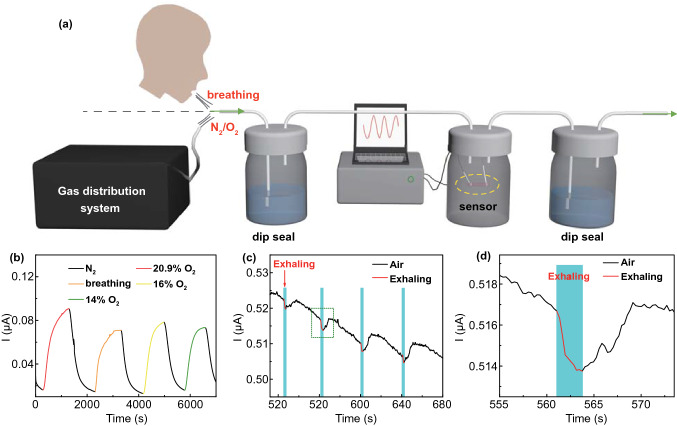
Application of the sensor in human respiration monitoring. **a** Schematic illustrating the setup for real-time detection of O_2_ concentration in human exhaled breath. **b** Dynamic response to 20.9% O_2_, exhaled breath, 16%, and 14% O_2_. **c** Real-time response to the gas exhaled by the human in the air. **d** Magnified image of the green box in **c**

Finally, we have proved the feasibility of applying the O_2_ sensor for real-life applications, such as the real-time detection of O_2_ concentration in human exhaled breath. We liquid-sealed the sensor with two plastic bottles filled with water at the front and back and then exploited the gas distribution system to feed N_2_ into the bottle where the sensor was located. Then, 20.9% O_2_, the gas exhaled by humans, 16% and 14% O_2_ were sequentially introduced into the bottle with a N_2_ background (Fig. 7a). Figure 7b shows that the Faraday current generated by human breath is between that produced by 16% and 14% O_2_, suggesting that the O_2_ content of the gas exhaled by the volunteer is about 15%, which is in line with the reality [67]. Considering the portability of the device, it is only necessary to replace the liquid sealing device with a small air bag to make it a portable system for monitoring human breath in practical application. It is worth noting that we have also deployed this O_2_ sensor to detect human breath directly in the air. To preclude the influence of humidity on O_2_ sensing, the sensor was simply covered by a sheet of hydrophobic Ecoflex to prevent the condensation of water on the organohydrogel (Fig. S17). Normally, the O_2_ concentration from exhaled breath (~ 15%) is lower than that in the air (~ 21%). Thereby, the O_2_ concentration around the sensor decreased when blowing the sensor with the mouth. Therefore, the current flowing through the sensor showed a downward trend. After stopping blowing, the O_2_ concentration around the sensor resumed, leading to the recovery of current to the original level (Fig. 7c, d). All these results manifest that this O_2_ sensor can be exploited to monitor human breath in real time.

Note that chitosan has been widely used in wound dressings because of its outstanding biocompatibility, biodegradation, antibacterial, and endogenous hemostasis properties [68]. The hydrophilic and bio-inert nature of PAM make it attractive for biomedical applications, among others [69]. Notably, appropriate O_2_ concentration in the wound tissue is essential for promoting wound healing [70]. Combining these characteristics and the self-adhesive property of the PAM-CS DN organohydrogel sensor, it can be potentially attached to the wound, not only for monitoring the O_2_ concentration at the wound in real-time but also for promoting the wound healing simultaneously. These advantages and application scenarios render the O_2_ sensor promising for wearable healthcare monitoring.

## Conclusions

In summary, we have successfully fabricated a stretchable, self-healable, self-adhesive, and high-performance O_2_ sensor based on PAM–CS DN organohydrogel, which was prepared through a facile soaking and solvent replacement strategy. Compared with the pristine hydrogel, the organohydrogel displays greatly enhanced mechanical strength, moisture retention, freezing resistance, and O_2_ sensitivity. The DN organohydrogel-based O_2_ sensor provides a full concentration detection range (0-100%), low LOD (5.7 ppm), sensitivity of 0.2%/ppm, linearity, selectivity, repeatability at RT, and the capability to work normally under a broad range of humidity and temperatures, which are competitive in comparison with that of existing O_2_ sensors. Attributing to the impressive deformability of organohydrogel, the sensors kept working under large deformations, such as 100% tensile strain, without degrading the sensing performance. Furthermore, both the sensitivity and the response/recovery speeds are boosted by applying strain. Notably, all the gas sensitivity, electrical conductance, and mechanical deformability of the organohydrogel are readily self-healed after being broken by an external force. In addition, due to the excellent self-adhesive property of organohydrogel within a broad range of temperatures, the O_2_ sensor can be directly adhered to various irregular substrates for working without demanding additional tapes. These unique attributes promote the development and application of the sensor in the field of wearable electronics.

Importantly, we have proposed the electrochemical reaction mechanism at the hydrogel-electrode interface to understand the O_2_ sensing behaviors of ion-conducting organohydrogel. This sensing mechanism was further corroborated via specially designed experiments. The practical applicability of this sensor in monitoring the O_2_ concentration from exhaled breath was also verified. Despite that only one type of organohydrogel was investigated here, its performance already exceeded that of conventional O_2_ transducing materials. This work opens the door to fabricating stretchable, self-healable, self-adhesive O_2_ sensors with high performance at RT using a family of ion-conducting organohydrogels as novel sensing materials for emerging wearable and healthcare applications.

## Supplementary Information

Below is the link to the electronic supplementary material.

Supplementary file1 (PDF 1985 KB)
