# Supplementary material for: Self-Healing, Self-Adhesive and Stable Organohydrogel-Based Stretchable Oxygen Sensor with High Performance at Room Temperature
Source: Nanomicro Lett. 2022 Jan 29;14:52. doi: 10.1007/s40820-021-00787-0 (PMC8800976; doi:10.1007/s40820-021-00787-0)
Supplement: Supplementary file 1 — Supplementary file1 (PDF 1985 KB) [file 40820_2021_787_MOESM1_ESM.pdf]

Supporting Information

Self-healing and Self-adhesive Organohydrogel based Stretchable Oxygen Sensor with High-performance at Room Temperature

Yuning Liang[1], Zixuan Wu[1], Yaoming Wei[1], Qiongling Ding[1], Meital Zilberman[3], Kai Tao[2], Xi Xie[1], and Jin Wu[1,*]

[1]State Key Laboratory of Optoelectronic Materials and Technologies and the Guangdong Province Key Laboratory of Display Material and Technology, School of Electronics and Information Technology, Sun Yat-sen University, Guangzhou 510275, China

[2]Ministry of Education Key Laboratory of Micro and Nano Systems for Aerospace, Northwestern Polytechnical University, Xi'an 710072, PR China

[3]Department of Biomedical Engineering, Faculty of Engineering, Tel Aviv University, Tel Aviv 69978, Israel

* Corresponding author. E-mail: wujin8@mail.sysu.edu.cn

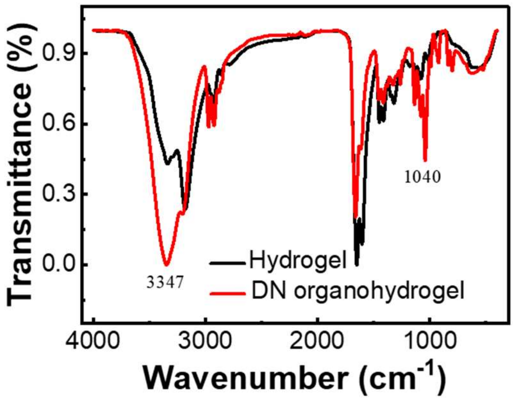

Fig. S1 FTIR spectra of polyacrylamide-chitosan hydrogel and the DN organohydrogel.

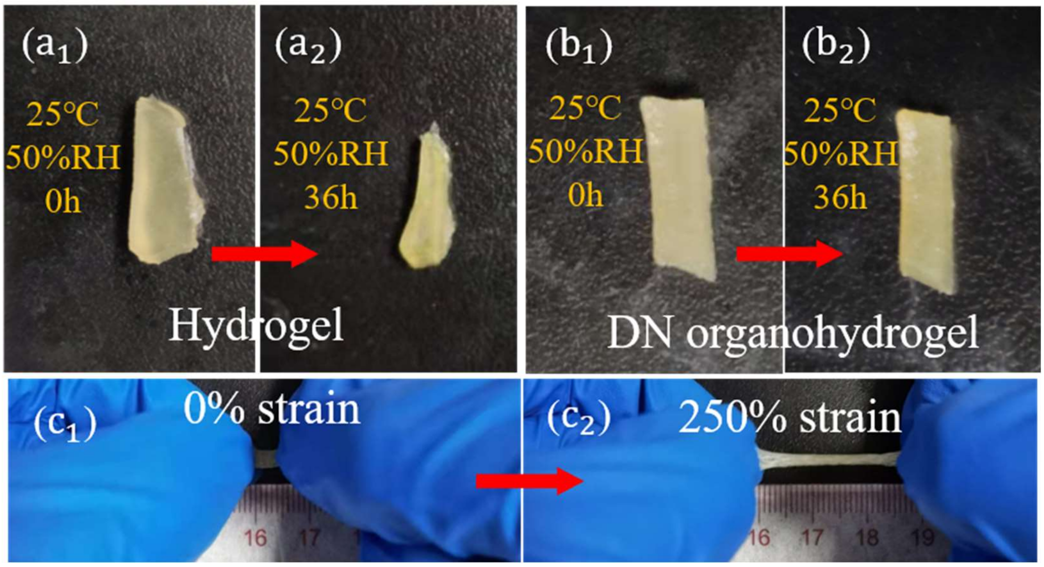

Fig. S2 a$_1$, a$_2$ Photographs of the untreated hydrogel before and after the drying experiment (storing at 24°C and 50%RH for 36 h), respectively. **b$_1$, b$_2$** Photographs of the DN organohydrogel before and after the same drying experiment, respectively. **c$_1$, c$_2$** Photographs of the DN organohydrogel at 0% and 250% tensile strains, respectively, after the drying experiment.

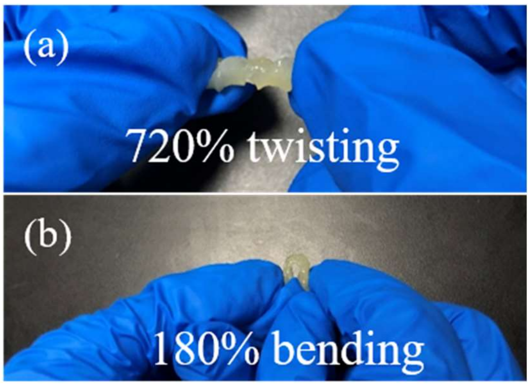

Fig. S3 Photographs showing the DN organohydrogel at **a** 720° twisting and **b** 180° bending, respectively.

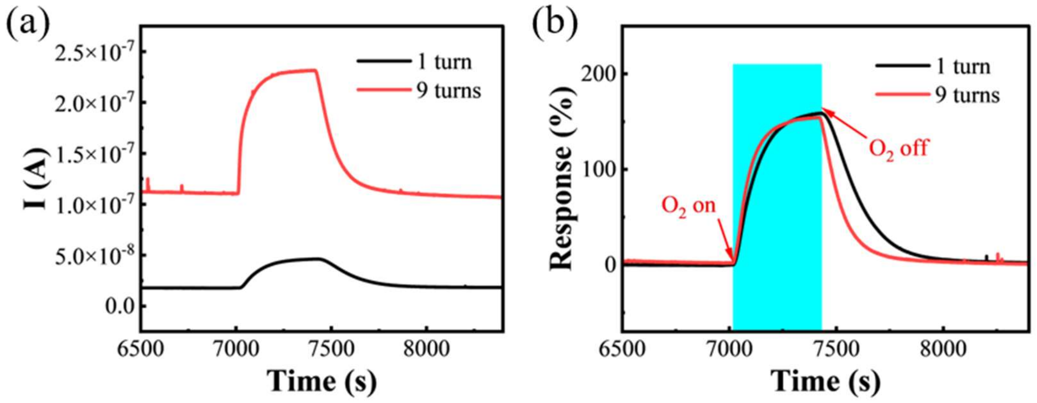

Fig. S4 The curves of **a** current and **b** dynamic responses to 0.4% O_2 versus time for the hydrogel oxygen sensors with one and nine turns of Ag coil electrode.

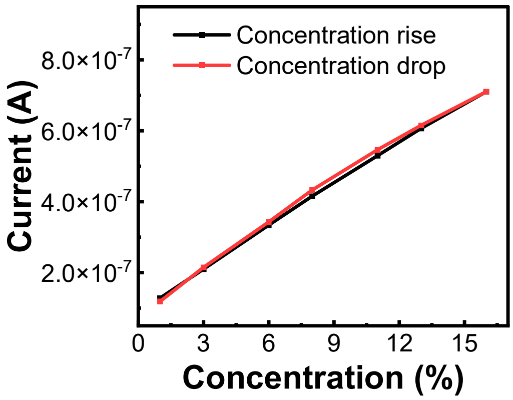

Fig. S5 Hysteresis curve of the oxygen sensor.

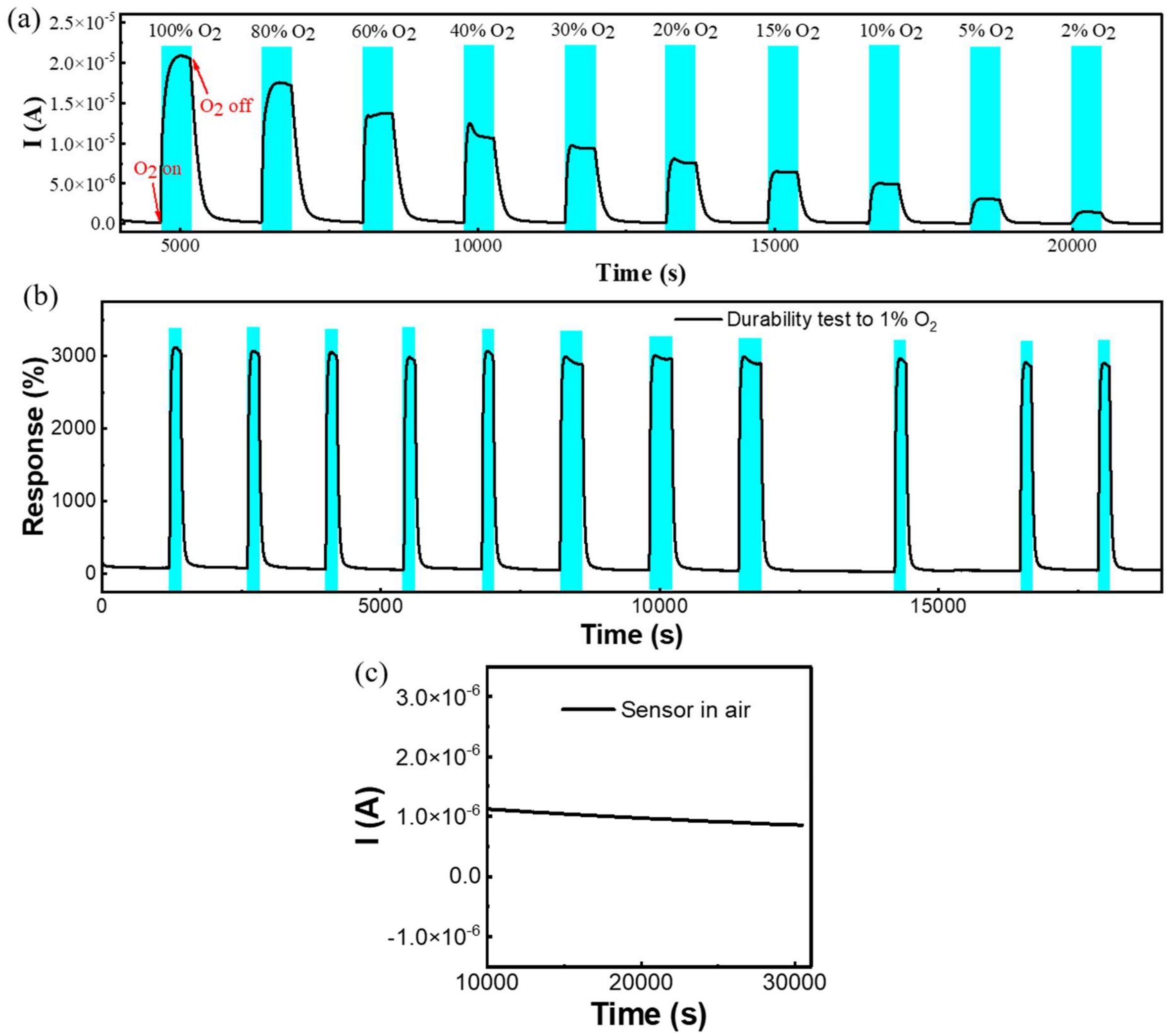

Fig. S6 a The curve of current versus time obtained by exposing the sensor to 2-100% O_2. **b** Dynamic and repeated response of the sensor to 1% O_2 within 6 h. **c** The curve of current versus time obtained by exposing the sensor to air for more than 8 h.

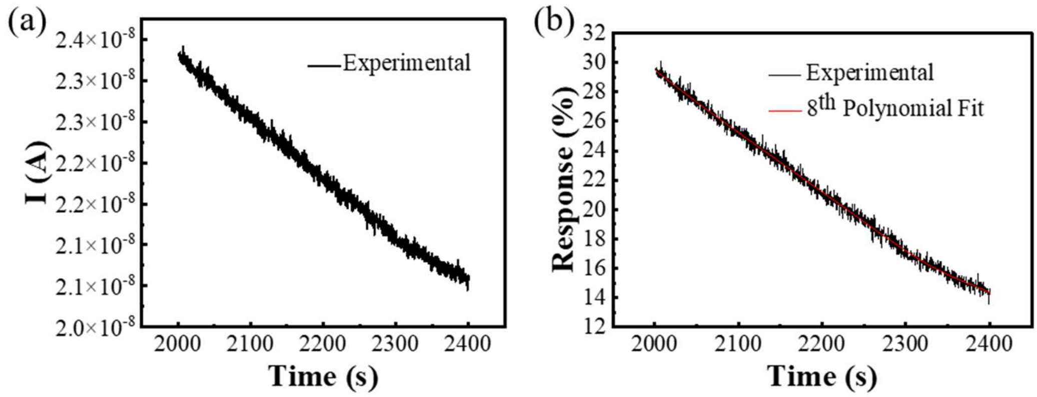

Fig. S7 Plots of the 5th order polynomial fitting graphs of **a** current and **b** response versus time of the sensor at the baseline before exposure to O_2.

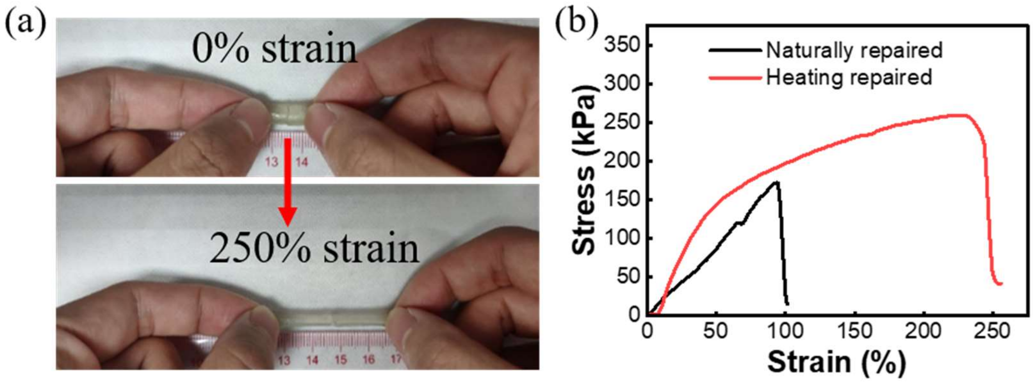

Fig. S8 a Photographs of the fractured DN organohydrogel at 0% and 250% strain after heating at 95°C for 10 min. **b** Stress-strain curves of the organohydrogels repaired naturally and by heating after breaking.

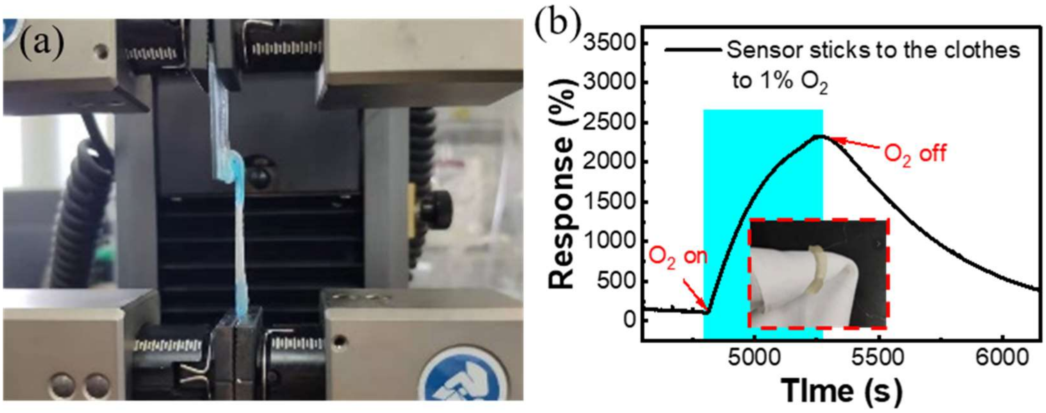

Fig. S9 a Photograph showing the setup for the peel strength testing. The peel strength was the average load per unit width of the bonding wire when the peel angle of the bonded material was 180°. It can be seen from Figure 3f that the peel force first increased rapidly with the displacement and then tended to be gentle, and its maximum value was the peel strength of the organohydrogel on the substrate. **b** Dynamic response of the stretchable organohydrogel-based sensor to 1% O_2 when attached to clothes. The photograph in the inset shows the self-adhesion of the stretchable sensor on the fabric for O_2 sensing without using an external adhesive.

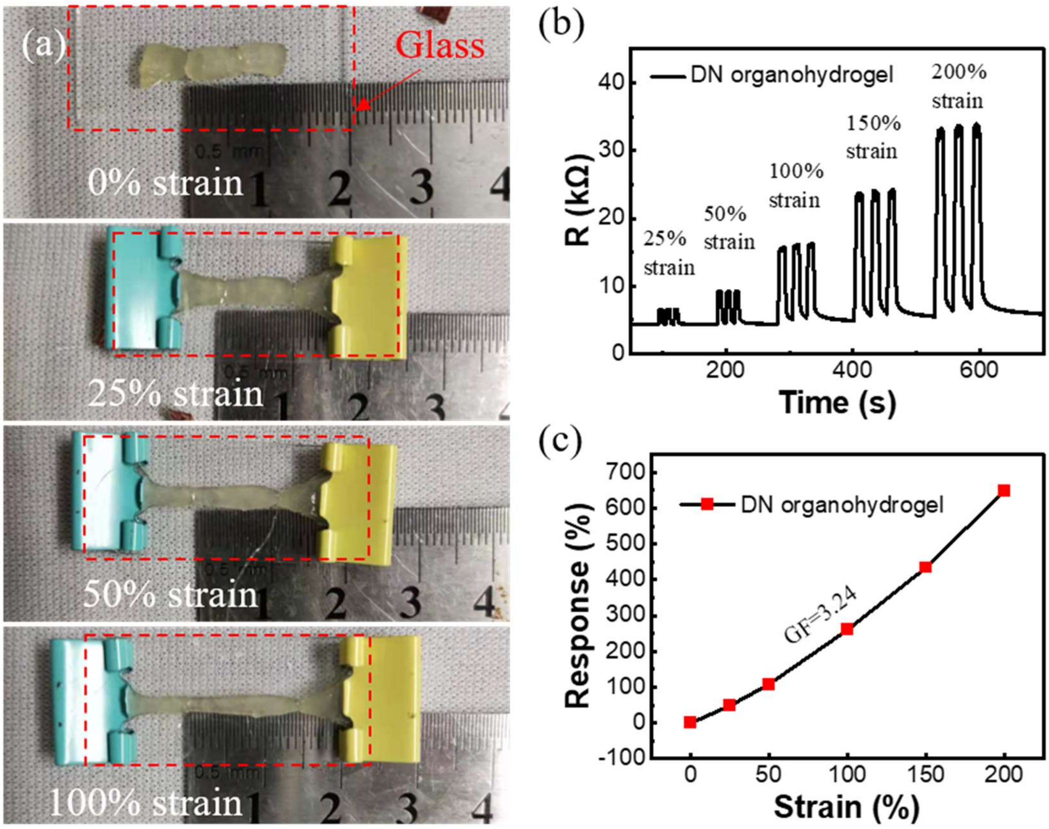

Fig. S10 a Photographs showing the gas-sensing test of the sensor at 0%, 25%, 50%, and 100% tensile strains. The red boxes denote the positions of transparent glass slide that was placed below the organohydrogel. **b** Time-dependent resistance variation of the DN organohydrogel sensor in response to 25%-200% tensile strains. **c** Plots of the relative resistance variation (response) of the sensor versus tensile strain. A GF value of 3.24 was derived from the linear fitting of the curve in **c**.

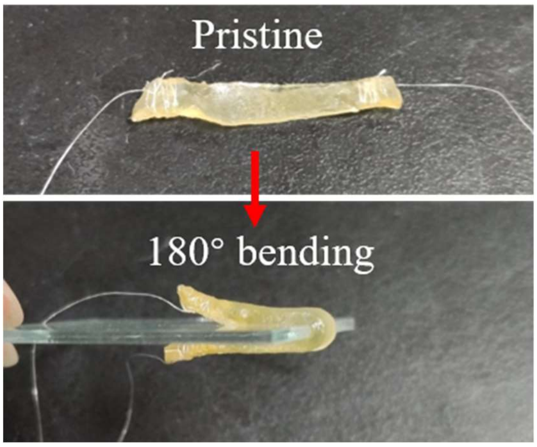

Fig. S11 Photograph of the gas-sensing test of the sensor in pristine and 180° bending states.

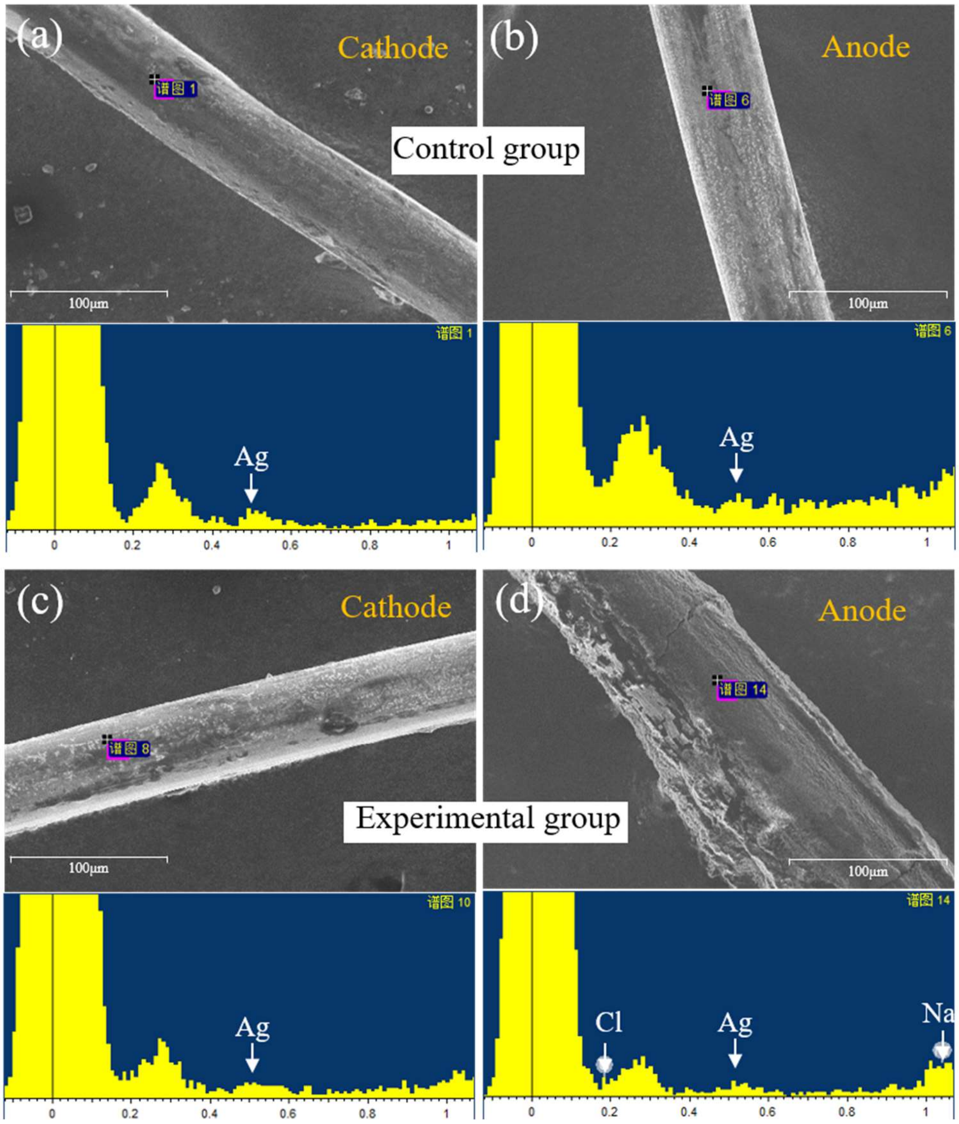

Fig. S12 The SEM images and EDS diagrams of **a** cathode and **b** anode in the control group, and **c** cathode and **d** anode in the experimental group, where above and below images were SEM image and corresponding EDS elemental spectra of the selected region in each figure. These results were obtained by exposing the sensors to air for 5 h with and without 5V DC voltage applied in the experimental and control groups, respectively.

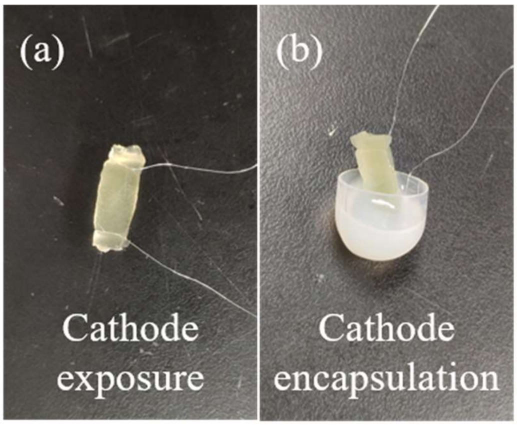

Fig. S13 Photographs of the sensor **a** before and **b** after cathode encapsulation.

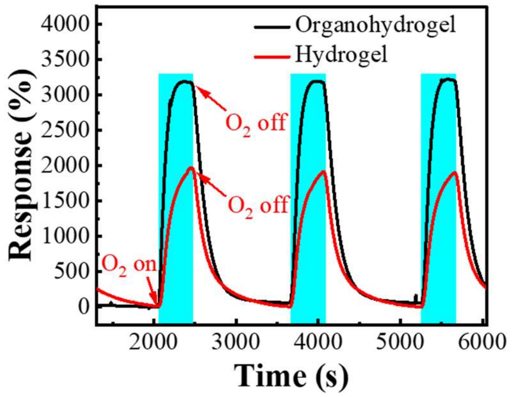

Fig. S14 Dynamic and repeated responses of the organohydrogel and hydrogel sensors to 1% O_2 for three cycles.

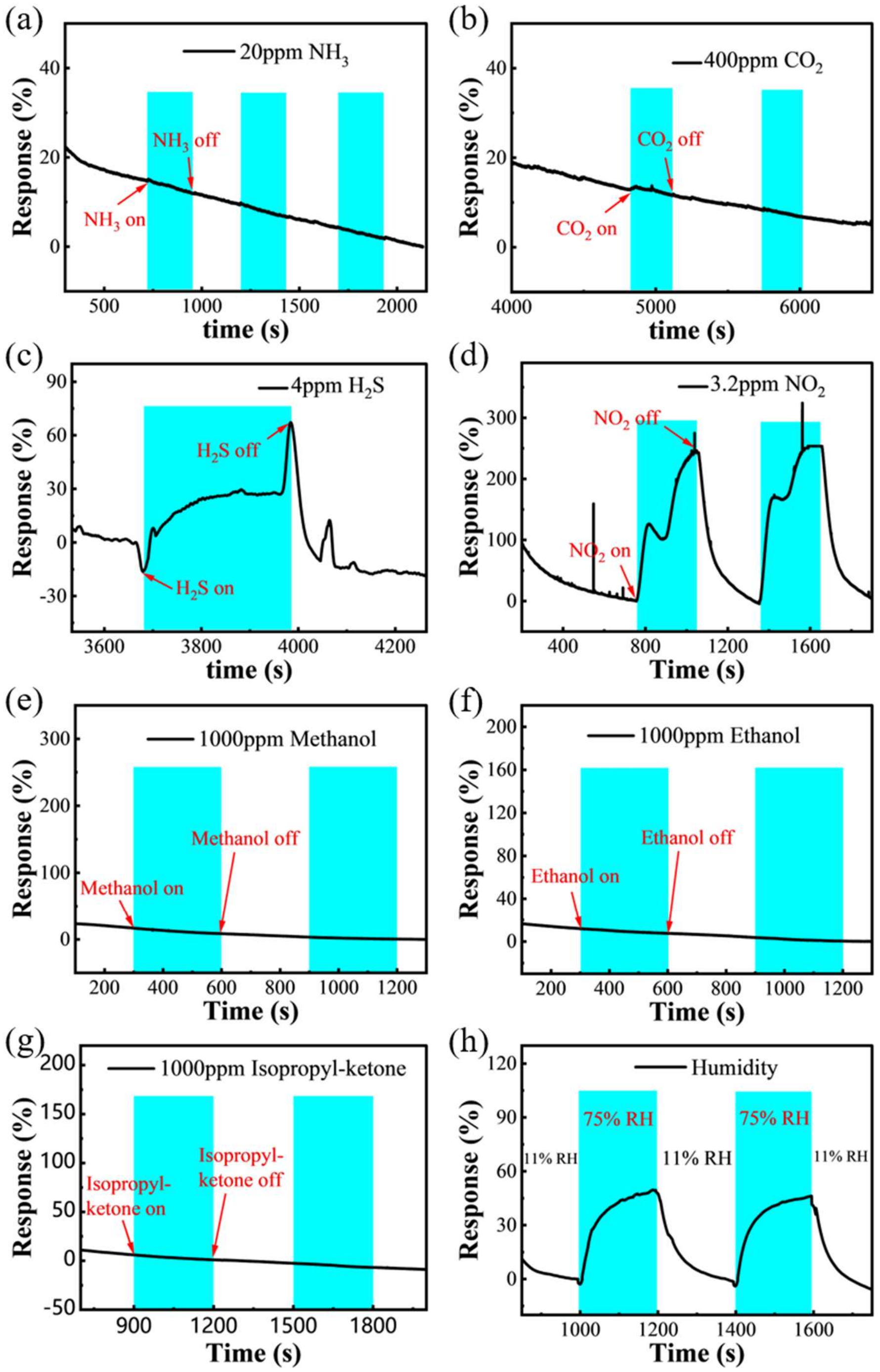

Fig. S15 Dynamic responses of the O_2 sensor to other gases, including **a** 20 ppm NH_3, **b** 400 ppm CO_2, **c** 4 ppm H_2S, **d** 3.2 ppm NO_2, **e** 1000 ppm methanol, **f** 1000 ppm ethanol, **g** 1000 ppm isopropyl-ketone, and **h** 75% RH.

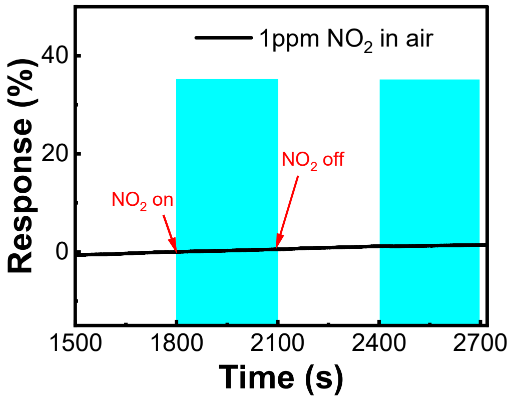

Fig. S16 Dynamic responses of the O_2 sensor to 1 ppm NO_2 in air environment.

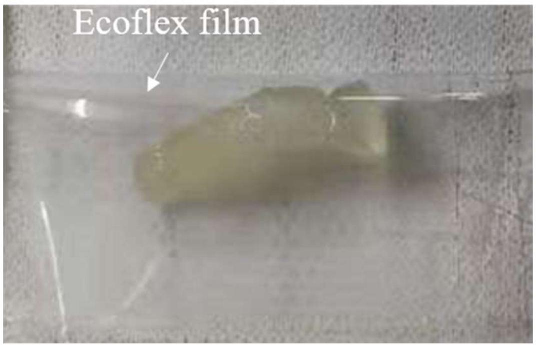

Fig. S17 The sensor was simply covered with a thin ecoflex film to preclude the interference of humidity for the detection of human breath directly.

Table S1. Comparison of the performances of oxygen sensors based on various sensing materials.

Sensing Material	Mechanical flexibility	Self-healing ability	O_2 detection range	Response/ Concentration	Response time [s]	Recover time [s]	Operating Temperature
MWCNTs (Thin Films)	Inflexible	No	0.3-100%	3.6%/5%	60	230	RT [1]
P-doped TiO_2	Inflexible	No	100-1000ppm	29.6/100ppm	35	20	116℃ [2]
Mn-doped ZnO	Inflexible	No	5-15ppm	3.8[a]/15ppm	150	90	RT [3]
LaOCl-doped SnO_2	Inflexible	No	100-5000ppm	2.25/250ppm	182	1315	RT [4]
MoS_2	Inflexible	No	1-100%	8.69/2%	130[a]	210[a]	300℃ [5]
PAM-CS DN Organohydrogel	≥1400% strain	Yes	0-100%	32/1%	40	64	RT[b]

[a] A value was not explicitly stated in the study, but approximated from a graphical plot; [b] present work.

Table S2. The 5th order polynomial fitting data used to calculate the LOD of the sensor at the baseline before exposure to O_2.

Time[s]	Y_i-Y	$(Y_i\text{-}Y)^2$
2000	-0.19358	0.037473216
2040	-0.02029	0.000411684
2080	-0.1246	0.01552516
2120	0.43442	0.188720736
2160	-0.12672	0.016057958
2200	0.08997	0.008094601
2240	-0.10502	0.0110292
2280	-0.26081	0.068021856
2320	0.11215	0.012577623
2360	0.32182	0.103568112
2400	0.074	0.005476

References

1. K. Rajavel, M. Lalitha, J. K. Radhakrishnan, L. Senthilkumar, R. T. Rajendra Kumar. Multiwalled carbon nanotube oxygen sensor: Enhanced oxygen sensitivity at room temperature and mechanism of sensing. ACS Appl. Mater. Interfaces. **7**, 23857-23865 (2015). https://doi.org/10.1021/acsami.5b04869

2. Z. Han, J. Wang, L. Liao, H. Pan, S. Shen, J. Chen. Phosphorus doped TiO_2 as oxygen sensor with low operating temperature and sensing mechanism. Appl. Surf. Sci. **273**, 349-356 (2013). https://doi.org/10.1016/j.apsusc.2013.02.041

3. F. Ahmed, N. Arshi, M. Anwar, R. Danish, B. H. Koo. Mn-doped ZnO nanorod gas sensor for oxygen detection. Curr. Appl. Phys. **13**, S64-S68 (2013).

4. Y. Xiong, W. Lu, D. Ding, L. Zhu, X. Li, C. Ling, Q. Xue. Enhanced room temperature oxygen sensing properties of laocl–SnO_2 hollow spheres by uv light illumination. ACS Sens. **2**, 679-686 (2017).

5. Y. H. Kim, K. Y. Kim, Y. R. Choi, Y.-S. Shim, J.-M. Jeon, J.-H. Lee, S. Y. Kim, S. Han, H. W. Jang. Ultrasensitive reversible oxygen sensing by using liquid-exfoliated MoS_2 nanoparticles. J. Mater. Chem. A **4**, 6070-6076 (2016). https://doi.org/10.1039/c6ta01277a
